# Efficient cellular solid-state NMR of membrane proteins by targeted protein labeling

**DOI:** 10.1007/s10858-015-9936-5

**Published:** 2015-05-09

**Authors:** Lindsay A. Baker, Mark Daniëls, Elwin A. W. van der Cruijsen, Gert E. Folkers, Marc Baldus

**Affiliations:** NMR Spectroscopy, Department of Chemistry, Faculty of Science, Bijvoet Center for Biomolecular Research, Utrecht University, Padualaan 8, 3584 CH Utrecht, The Netherlands; Oxford Particle Imaging Centre, The Wellcome Trust Centre for Human Genetics, Division of Structural Biology, Nuffield Department of Medicine, University of Oxford, Roosevelt Drive, Oxford, OX3 7BN UK

**Keywords:** Solid-state NMR, Membrane protein, Cellular membranes, Protein expression

## Abstract

Solid-state NMR spectroscopy (ssNMR) has made significant progress towards the study of membrane proteins in their native cellular membranes. However, reduced spectroscopic sensitivity and high background signal levels can complicate these experiments. Here, we describe a method for ssNMR to specifically label a single protein by repressing endogenous protein expression with rifampicin. Our results demonstrate that treatment of *E. coli* with rifampicin during induction of recombinant membrane protein expression reduces background signals for different expression levels and improves sensitivity in cellular membrane samples. Further, the method reduces the amount of time and resources needed to produce membrane protein samples, enabling new strategies for studying challenging membrane proteins by ssNMR.

**Electronic supplementary material:**

The online version of this article (doi:10.1007/s10858-015-9936-5) contains supplementary material, which is available to authorized users.

## Introduction

Membrane proteins (MPs) are often challenging to study by conventional structural biology methods and their native environment, a cellular membrane, is very heterogeneous and difficult to replicate in vitro. Although many membrane mimetics are available, the choice of mimetic can alter the biophysical properties of MPs (see, for example, Zhou and Cross 2013; Etzkorn et al. 2013). This phenomenon is one reason for the recent push towards crystallizing MPs in lipidic mesophases (Caffrey et al. 2012, for a review).

Solid-state nuclear magnetic resonance spectroscopy (ssNMR) is one method available to study the structure and dynamics of MPs in a lipid bilayer. ssNMR has been used successfully to determine the structure of membrane proteins (see, for example, Andronesi et al. 2005; Das et al. 2012; Wang et al. 2013a; van der Cruijsen et al. 2013), as well as investigating molecular interactions (Miao and Cross 2013, for a review) and function (Baker and Baldus 2014, for a review). However, most of these studies have used synthetic lipid bilayers, which can mimic the physical environment of cellular membranes but often lack their chemical heterogeneity. In addition to more closely resembling the cellular environment, the use of native membranes can remove the need to purify the MP of interest, eliminating any potential for structural disruptions during the solubilization process and speeding up sample preparation.

Several groups have demonstrated that ssNMR can be used to study structure and dynamics of proteins embedded in native lipid membranes (Etzkorn et al. 2007; Fu et al. 2011; Jacso et al. 2012; Miao et al. 2012; Kulminskaya et al. 2012; Ward et al. 2015). Previous work has demonstrated that it is possible to study MPs in cellular envelopes and whole cells of *Escherichia coli* by conventional (Renault et al. 2012b; Kaplan et al. 2015) and dynamic nuclear polarization (DNP)-enhanced ssNMR (Renault et al. 2012a; Yamamoto et al. 2014; Kaplan et al. 2015). In particular, we could show that studies using cellular envelopes or whole cells of Gram-negative bacteria are facilitated by use of a special *E. coli* deletion strain, to remove signals from naturally highly abundant outer MPs, i.e., OmpA and OmpF. In these preparations, signals from other cellular components, such as lipids, nucleotides, peptidoglycan and lipopolysaccharides, remain visible at intensities similar to that of the (overexpressed) protein of interest (Baldus 2015). Such non-proteinaceous correlations can help to refine the supramolecular structure of a protein in a membrane setting (see, e.g., Weingarth and Baldus 2013). Further, these signals can be used to study the structure of other molecular components such as RNA (Renault et al. 2012a) or material in the cell walls of bacteria and plants (Dick-Pérez et al. 2011, 2012; Wang et al. 2012, 2014), as well as helping to clarify the cellular distribution of added reagents, such as radicals for DNP experiments (Takahashi et al. 2013).

On the other hand, these cellular signals can complicate the analysis of spectra of MPs that have not previously been characterized in synthetic bilayers. To reduce the challenges posed by the high background levels in cellular membrane samples, it would be beneficial to have a system by which isotope labels could be selectively incorporated into a MP of interest. Here, we demonstrate that treatment of cells with the antibiotic rifampicin, to inhibit the native RNA polymerase, produces native MP samples with significantly less background in ssNMR spectra. Previously, this concept was used for preparation of labeled proteins for solution-state NMR without purification (Almeida et al. 2001) with potential benefits to prevent cell lysis in the context of in-cell solution-state NMR experiments (Cruzeiro-Silva et al. 2006). During rifampicin treatment, the *E. coli* RNA polymerases are inhibited, preventing endogenous gene expression. However, systems for over-expression of proteins in *E. coli*, such as the T7 system, often use non-*E. coli* RNA polymerases that are not inhibited by rifampicin. If one induces expression of the heterologous protein after rifampicin treatment, the heterologous protein should be produced while endogenous protein production should decline. The suppression of endogenous protein production should direct any isotopically labeled nutrients towards heterologous protein production, hopefully resulting in an isotopically labeled target protein within a natural abundance cellular background. By coordinating the inhibition of endogenous gene expression with exposure to isotopically labeled media and induction of heterologous protein expression, rifampicin could provide a means to specifically isotopically label a heterologously expressed protein.

Using membrane proteins involved in protein insertion (YidC) with a high level of recombinant expression and ion transport (KcsA) exhibiting low level of recombinant expression, we show that this approach can be used to target labels to proteins of interest, resulting in spectral simplification and decreased isotope labeling costs. Further, the method also can be adapted to suit different labeling schemes and protein expression levels, and can be used efficiently even when the amount of recombinant protein is significantly less than that of major endogenous MPs [which have copy numbers of ~10^5^/cell (Renault et al. 2012b and references therein)]. By removing the need for high expression levels and purification of MPs, this method expands the applicability of ssNMR experiments to wider range of challenging MPs.

## Methods

### Cloning and recombinant protein expression

The gene for YidC from *Escherichia coli* (UniProt ID: P25714**)** was cloned into pHisLic vector with an N-terminal 6× histidine tag for expression under a T7 system using enzyme-free cloning, as described previously (de Jong et al. 2006). The gene for KcsA from *Streptomyces lividans* (UniProt ID: P0A334) was obtained in a pT7 vector, as described previously (van Dalen et al. 2000). *E. coli* LEMO21 cells (New England Biolabs) were transformed with these plasmids by heat shock before plating on LB with ampicillin (AMP) at 50 μg/mL and chloramphenicol (CAM) at 35 μg/mL. Cells were cultured according to standard practice (see also Baker et al. 2015). Precultures were grown in LB with AMP and CAM and then transferred to M9 minimal media (Folkers et al. 2004) supplemented with 1.0 g/L ^14^NH_4_Cl, 5.0 g/L ^12^C_6_-glucose, AMP and CAM overnight. Between 50 and 500 mL volumes of M9, supplemented as above, were inoculated with the overnight cultures to OD 0.1, and grown at 37 °C with shaking until reaching an OD ~1.5–2.0. Cells were harvested by centrifugation at 4000×*g* for 15 min before resuspension in equal volumes isotopically labeled supplemented M9 (1.0 g/L ^15^NH_4_Cl, 5.0 g/L ^13^C-glucose for uniformly labeled samples; 1.0 g/L ^14^NH_4_Cl, 5.0 g/L ^12^C-glucose and 200 mg/L each ^15^N and ^13^C labeled amino acid for specifically labeled samples). IPTG (isopropyl β-d-1-thiogalactopyranoside) at 0.5–1 mM final concentration was added to induce expression of the T7 polymerase and the cultures were incubated at 28 °C for 30 min with shaking. Rifampicin was added to a final concentration of 100 μg/mL and the cultures were incubated with shaking in the dark at 28 °C overnight. Cells were harvested by centrifugation at 4000×*g* for 15 min at 4 °C, and resuspended in 5–10 mL cold lysis buffer (20 mM Tris pH 7.4, 100 mM NaCl).

### Cellular membrane isolation, inner membrane purification, and ssNMR sample preparation

Cells were lysed in a pressure cell homogenizer (Stansted) at 8000 psi without the addition of lysozyme. Cell debris was removed by centrifugation at 7000×*g* for 15 min, and any inclusion bodies were removed, where necessary, by centrifugation at 25,000×*g* for 15 min. Membranes were harvested by centrifugation at 100,000×*g* for 1 h.

Inner and outer membrane fractions were separated with sucrose gradients (Baker et al. 2015). Briefly, 2 mL of 55 % (w/v) sucrose, 8 mL of 51 % (w/v) sucrose, 8 mL of 45 % (w/v) sucrose, and 5 mL of 36 % (w/v) sucrose, all in 50 mM Tris pH 8.0 buffer, were poured in a stepwise manner, with 4 mL of sample mixed with sucrose to a final concentration of 20 % (w/v) layered on top. The gradients were centrifuged overnight in a SW32-Ti swinging bucket rotor (Beckman) at 100,000×*g*. Fractions corresponding to the outer membranes, inner membranes, and a mixture of outer and inner membranes were harvested with a syringe at the interface between the 55 and 51, 45 and 36, and 51 and 45 % sucrose layers, respectively. The band of mixed outer and inner membranes was observed after growth in M9 media, but not after growth in LB.

Membranes were washed twice with buffer (10 mM phosphate buffer pH 6.8 for YidC; 10 or 50 mM phosphate buffer pH 7.0, 10 or 50 mM potassium chloride, 10 or 50 mM sodium chloride, and 0.01 % sodium azide for KcsA inner and cellular membrane preparations, respectively) and collected by centrifugation at 125,000×*g* for 1– 2 h before packing into a 3.2 mm rotor for magic angle spinning (MAS).

### Purification of reference proteoliposome samples

YidC with a hexa-histidine tag was over-expressed for purification as described for the cellular samples, with the following differences. After preculture, the main culture was inoculated in M9 with ^13^C_6_-glucose and ^15^NH_4_Cl at OD 0.1, and grown to OD 0.6 prior to induction with IPTG and 10 μM final concentration rhamnose. Rhamnose treated cells were found to produce more YidC when induced at lower ODs; no improvement was observed for induction at higher ODs like those used for rifampicin treatment. Cells were grown at 37 °C for a further 3–5 h until the OD was greater than 2.0. Cells were pelleted and lysed, and the membranes isolated as described for the cellular NMR samples. Membranes were solubilized at 4 °C by stirring with 2 % (w/v) dodecyl maltoside (DDM) (Anatrace) in 10 mM phosphate buffer (pH 6.8) containing 250 mM NaCl, 2 mM 2-mercaptoethanol, 10 % (v/v) glycerol, 0.03 % (w/v) DDM and 20 mM imidazole (Buffer A) for 2 h. Insoluble material was pelleted at 100,000×*g* for 1 h, and the supernatant was bound to 5 mL Ni–NTA resin (Qiagen) overnight. The resin was washed with 4 column volumes (CV) of buffer A, and then with 2 CV of buffer B (10 mM phosphate buffer pH 6.8, 100 mM NaCl, 2 mM 2-mercaptoethanol, 10 % (v/v) glycerol, 0.03 % (w/v) DDM and 20 mM imidazole). YidC was eluted with 5 CV of buffer B with 380 mM additional imidazole. The eluent was dialyzed four times for ~2 h against 75 mL of Buffer B without imidazole. The protein concentration was estimated using the BCA assay (Pierce) and then mixed with *E. coli* polar lipid extract (Avanti Polar Lipids), dissolved in water, at a ratio of 2 mg YidC: 1 mg lipid. DDM was removed by overnight incubation with BioBeads (BioRad) and YidC proteoliposomes were harvested by centrifugation at 100,000×*g* for 1 h. The proteoliposomes were washed in 10 mM phosphate buffer, pH 6.8, and pelleted at 125,000×*g* for ~2 h before being packed into a 3.2 mm MAS rotor.

KcsA was over-expressed, purified, and reconstituted as described previously (Lange et al. 2006; van der Cruijsen et al. 2013).

### Spectroscopy, processing, and referencing

All YidC samples were measured on a 700 MHz narrow-bore spectrometer (Bruker Biospin, Germany) with a 3.2 mm ^1^H, ^13^C, ^15^N MAS probe at 13 kHz MAS frequency, unless otherwise noted, and with a set temperature of 258 K (corresponding to an effective temperature of ~4 °C). Hartmann–Hahn ^1^H–^15^N and ^1^H–^13^C cross polarization (CP) used contact times of 650 and 500 μs, respectively, and 70–100 % ramps. ^15^N–^13^C SPECIFIC CP (Baldus et al. 1998) used a contact time of 4500 μs with optimized irradiation frequencies of 30.3 and 19 kHz, for ^15^N and ^13^C, respectively, and a ramp of 90–100 %. PARIS (Weingarth et al. 2009) was used for ^13^C–^13^C mixing, with a mixing time of 30 ms, 7.3 kHz ^1^H irradiation, and MAS frequency of 11 kHz. In all experiments, decoupling used SPINAL64 (Fung et al. 2000) with ^1^H irradiation of 83 kHz. ^15^N-edited ^13^C–^13^C detected experiments used Hartmann–Hahn ^1^H–^15^N CP followed by ^15^N–^13^C_α_ SPECIFIC CP and PARIS mixing.

KcsA samples were measured on an 800 MHz wide-bore spectrometer (Bruker BioSpin, Germany) with a 3.2 mm ^1^H, ^13^C, ^15^N MAS probe. Experiments were recorded at a set temperature of 253 K and MAS frequency of 12 kHz. CP contact times were 400 μs with a ramp of 70–100 % for ^1^H–^15^N, SPECIFIC CP used 3000 μs contact time with 90–100 % ramp, and irradiation of 57, 38, and 29 kHz was used for ^1^H, ^15^N, and ^13^C pulses, respectively. Decoupling used SPINAL64 with 83.3 kHz irradiation.

Spectra were referenced against adamantane (Harris et al. 2008) and histidine (Wei et al. 1999) powders. Data were processed with TopSpin 3.0 (Bruker Biospin) and analyzed using Sparky (Goddard and Kneller 2008). Chemical shift predictions were made with Shiftx2 (Neal et al. 2003) and FANDAS (Gradmann et al. 2012) using atomic model PDB ID 3wvf (Kumazaki et al. 2014).

## Results and discussion

**Fig. 1:**
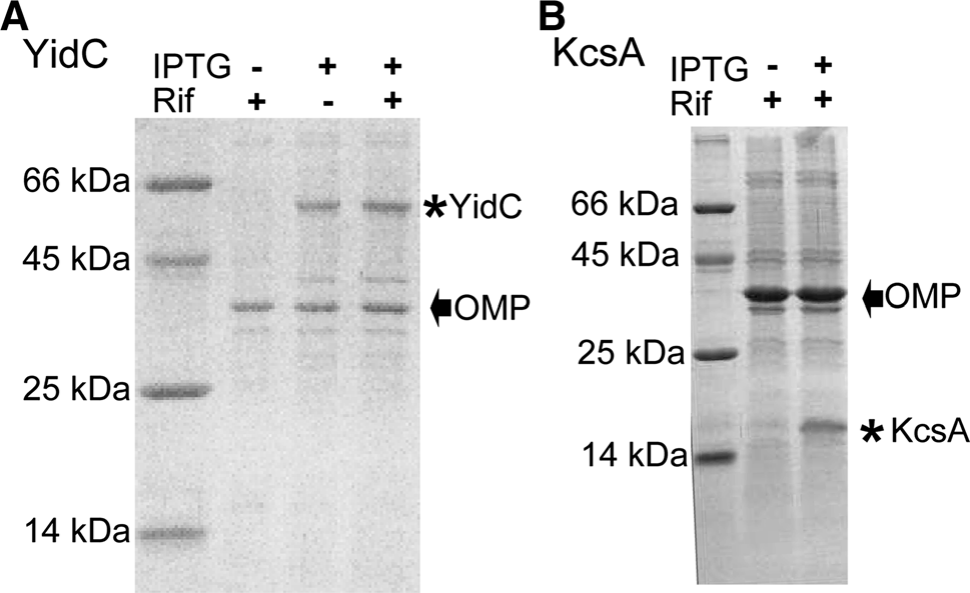
Rifampicin can be used to selectively over-express membrane proteins. YidC and KcsA were overexpressed in *E. coli* LEMO cells during treatment with rifampicin, as seen by SDS-PAGE of crude cell lysate on a 12.5 and 13.75 %, respectively, acrylamide gel. **a** YidC: *First lane* marker; *second lane* without induction (−IPTG) and with rifampicin (+Rif) treatment; *third lane* with induction (+IPTG) but without rifampicin (−Rif); *fourth lane* with induction (+IPTG) and rifampicin (+Rif). **b** KcsA: *First lane* marker; *second lane* without induction (−IPTG) and with rifampicin (+Rif) treatment; *fourth lane* with induction (+IPTG) and rifampicin (+Rif). The band corresponding to YidC in **a** and KcsA in **b** is marked (*asterisk*), as is the band corresponding to the highly naturally abundant outer membrane proteins (OMP). OmpF and OmpA would run at this expected molecular weight, however, there is some overlap between OMPs (Renault et al. 2012b)

To study the effect of adding rifampicin during cellular protein production and to assess the influence of ssNMR signals stemming from background molecules on our spectra, we conducted a series of ssNMR experiments using different sample preparation strategies for the case of high (YidC) and low (KcsA) protein expression. As a first target, we selected YidC, an ~61 kDa inner membrane protein from *E. coli*, that could be overexpressed during rifampicin treatment at sufficient levels to be observed clearly by SDS-PAGE (denaturing polyacrylamide gel electrophoresis) in crude cell lysate (Fig. 1a). On the other hand, expression of the tetrameric 64 kDA KcsA channel was visible but significantly lower (Fig. 1b) compared to the band corresponding to the highly naturally abundant (Renault et al. 2012b) outer membrane proteins OmpF and OmpA (denoted as OMP in Fig. 1a, b). SDS-PAGE will show all proteins present in the cellular membrane sample, not just those that are isotopically labeled. Therefore, to establish whether the targeted isotopic labeling was successful, we examined the cellular membrane samples by ssNMR.

**Fig. 2:**
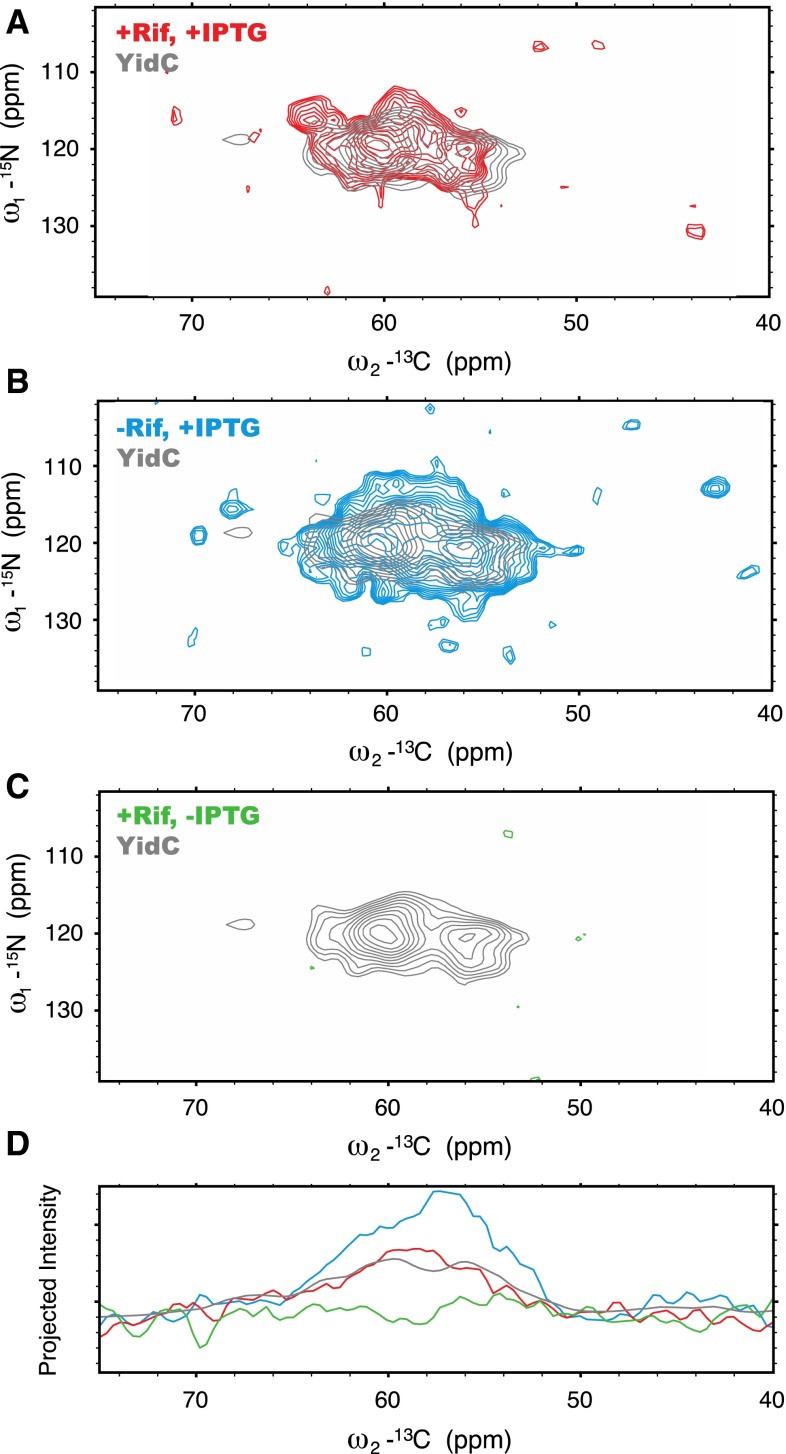
^15^N-^13^C_α_ CP-based correlation spectra of YidC in **a** cellular membranes with IPTG and rifampicin (*red*), **b** cellular membranes without rifampicin but with IPTG (*blue*), and **c** cellular membranes with rifampicin and without IPTG (*green*). The spectrum of YidC after purification and reconstitution into *E. coli* lipid extract proteoliposomes is shown in *grey* in each panel with the same processing as the cellular membrane samples. All spectra were collected with a 350 μs increment for 6 ms acquisition time in the indirect dimension and 8 ms acquisition in the direct, averaged over 1072 scans, and processed after zero-filling without linear prediction with 150 Hz line broadening. The lowest contour levels were set as ~2.7× the standard deviation of the noise in the cellular membrane samples. The lowest contour level for the spectrum of purified YidC was chosen to keep the number and spacing of contours the same as for the cellular membrane samples. **d** Each spectra in **a**–**c** was projected onto the ^13^C plane in order to compare total signal between samples with and without rifampicin and IPTG. *Red* with IPTG and rifampicin; *blue* with IPTG and without rifampicin; *green* without IPTG and with rifampicin; *grey* purified YidC, scaled to the same range as the spectrum of cellular membranes with rifampicin and IPTG (*red*)

We first examined fully (^13^C,^15^N) isotopically labeled YidC in the cellular membrane samples prepared with rifampicin and IPTG by ssNMR in a ^15^N–^13^C_α_ correlation CP-based spectrum (Fig. 2a, red) and compared our data to results obtained using purified YidC proteoliposomes (Fig. 2a, grey). Approximately 50 mL of culture at OD 2.0 was needed to fill a 3.2 mm MAS rotor with isolated cellular membranes. Using large exponential line broadening during processing, we obtained an overall insight into the NCA signatures of purified YidC in reference to the cellular sample with rifampicin. Within the resolution expected for a 548 amino-acid membrane protein, both preparations resulted in similar spectra. Subsequently, we prepared YidC cell envelopes without rifampicin (Fig. 2b, blue), to elucidate the effect of background signals on the interpretation of the cellular membrane samples. Without rifampicin, additional signals were observed. The difference in signal can clearly be seen by comparing projections of the ^15^N–^13^C_α_ correlation spectrum (Fig. 2d). These additional signals can be attributed to isotopic labels incorporated in a non-specific manner, reflecting the diversity of proteins being produced during induction and continued cell growth. Finally, we prepared samples with rifampicin but without IPTG, to determine the amount of background signals actually present in the rifampicin-treated cellular membrane preparations (Fig. 2c, green). In this case, only a very low level of background was detected. The spectrum for each of the cellular membrane samples was recorded and processed with identical conditions. Taken together, these results suggest that the rifampicin treatment improves the detection of YidC in a cellular membrane context in ^15^N–^13^C_α_ correlation spectra.

**Fig. 3:**
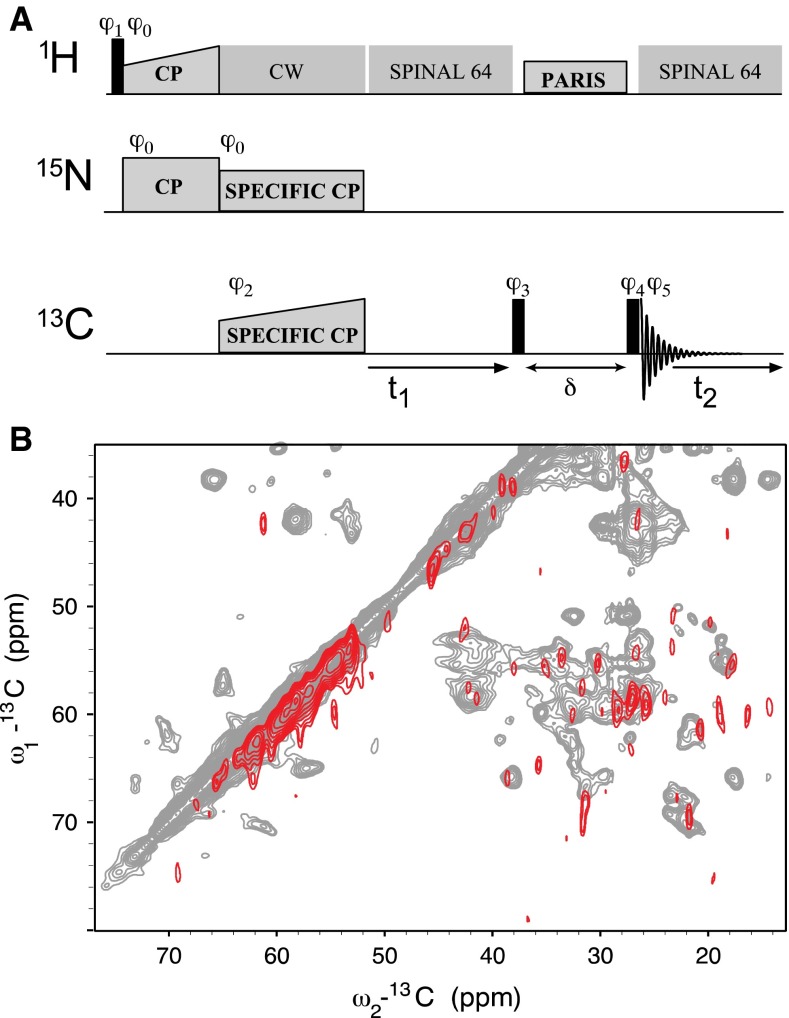
**a** Schematic diagram of pulse sequence used for CP-based ^15^N-edited ^13^C–^13^C correlation spectra. After CP-based transfer from ^1^H to ^15^N, Specific CP is used to transfer to ^13^C_α_ (with field offset Δω), followed by a PARIS mixing block of length δ. Continuous wave (CW) and SPINAL 64 decoupling schemes were used as indicated. The phase cycling follows φ_0_ = (0), φ_1_ = (13), φ_2_ = (0022), φ_3_ = (3), φ_4_ = (0000 1111 2222 3333), φ_5_ = (0220 1331 2002 3113). **b** A ^13^C–^13^C correlation spectrum of fully isotopically labeled membrane protein YidC (*red*) was recorded with ^15^N editing to remove signal from lipids. A CP-based ^15^N-edited ^13^C–^13^C PARIS (30 ms mixing) correlation spectrum of YidC in rifampicin cellular membranes was collected with a 114 μs increment for 2.5 ms total acquisition time in the indirect dimension and 10 ms acquisition in the direct, averaged over 2800 scans, and processed after zero-filling without linear prediction with a line broadening of 75 Hz. This experiment was extremely insensitive in comparison to a CP-based ^13^C–^13^C PARIS (30 ms mixing) correlation spectrum of purified YidC and *E. coli* lipid proteoliposomes, overlaid in *grey* as a reference

The potential for the use of ^13^C–^13^C correlation spectra also was explored; however, due to the high levels of ^13^C-labeled phospholipids in the fully labeled cellular membrane samples, we performed ^15^N-edited ^13^C–^13^C correlation CP-based experiments in which SPECIFIC CP transfer (Baldus et al. 1998) from ^15^N to ^13^C_α_ nuclei is followed by a PARIS mixing block to detect aliphatic C_α_–C_x_ correlations in a two-dimensional (^13^C, ^13^C) correlation experiment (Fig. 3a). In Fig. 3b, results of such an experiment for YidC (red) are compared to data obtained using a non-edited ^13^C–^13^C correlation CP-based experiment with reconstituted YidC liposomes (grey). Although the spectral overlap was reduced compared to the ^15^N–^13^C_α_ spectra, the sensitivity of the ^15^N-edited experiment was limited, possibly due to signal loss during the additional magnetization transfer step in comparison to non-^15^N-edited experiments.

**Fig. 4:**
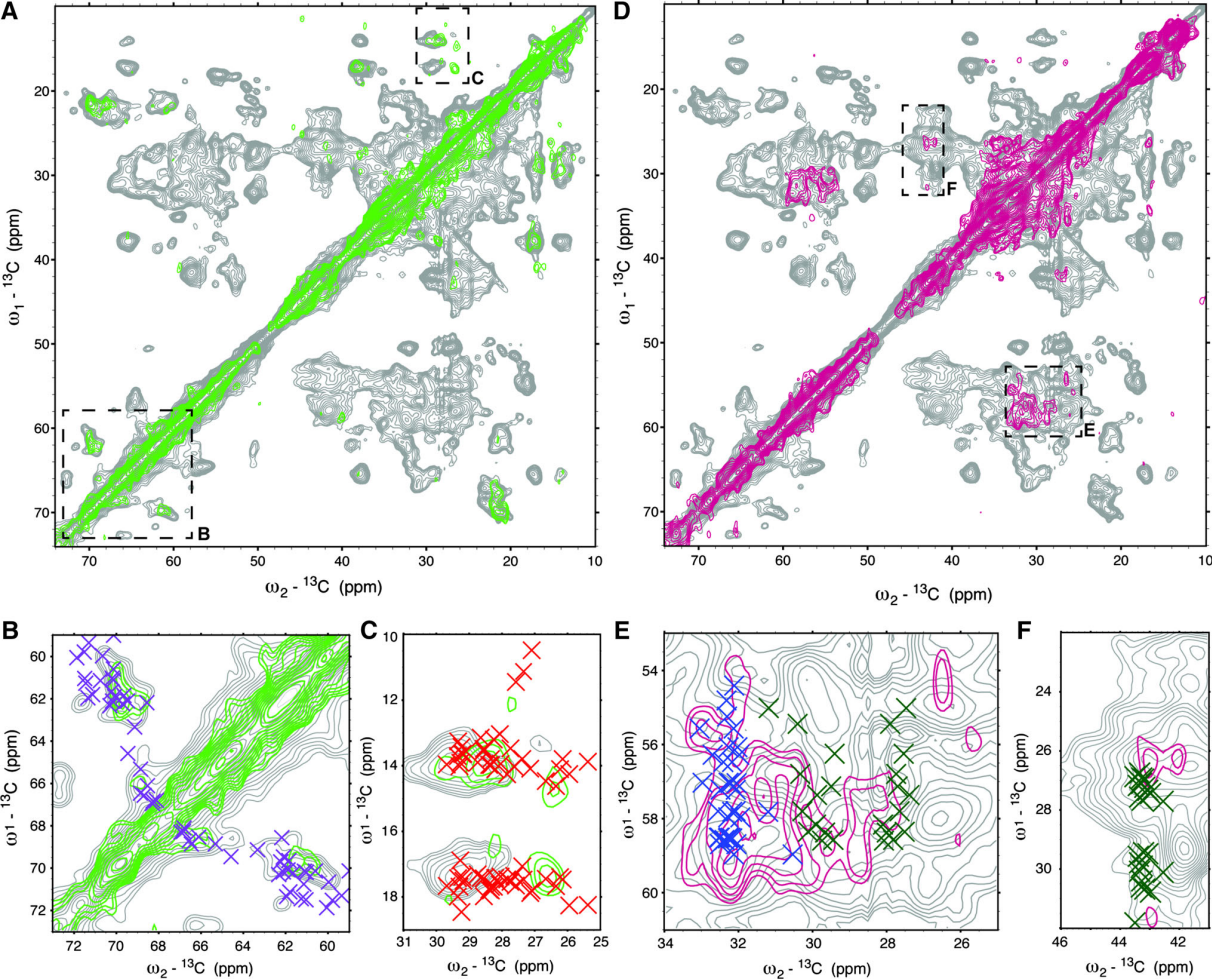
Spectra of large membrane proteins can be improved with specific amino acid labeling. **a**, **d** CP-based ^13^C–^13^C PARIS (30 ms mixing) correlation spectra of YidC in rifampicin cellular membranes that are threonine and isoleucine labeled (*green*), and methionine and arginine labeled (*pink*). In both spectra, a CP-based ^13^C–^13^C PARIS (30 ms mixing) correlation spectra of purified YidC in *E. coli* lipid proteoliposomes is overlaid in *grey*. **b**, **c** Magnified views of the boxed areas in **a**. Although threonine (predicted resonances shown as* purple crosses*) and isoleucine (predicted resonances shown as *red crosses*) comprise only ~13 % of the residues in YidC, there is sufficient resolution and sensitivity in ^13^C CP-based spectra to separate threonine residues with alpha helical and beta-strand secondary structure. **e**, **f** Magnified views of the boxed areas in **d**. Methionine predicted resonances are shown as* blue crosses* and arginine predicted resonances as *green crosses*. In the Met–Arg labeled cell envelopes, only ~6 % of residues are labeled, and hence the sensitivity is less than that of the Thr–Ile labeled. The spectra were recorded with a 40 μs increment and 3 and 8 ms total acquisition time in the indirect and direct dimensions, respectively, averaged over 176 and 272 scans for (**a**–**c**) and (**d**–**f**), respectively, and processed after zero-filling without linear prediction with a line broadening of 75 Hz. Predicted correlations were calculated using ShiftX (Neal et al. 2003) and FANDAS (Gradmann et al. 2012) based on the recent crystal structure of YidC from *E. coli* [PDB ID 3wvf (Kumazaki et al. 2014)]

To further reduce spectral contributions from non-proteinaceous cellular components for samples prepared with ^13^C glucose, we used the rifampicin treatment protocol to label specific amino acids, as described in "Materials and methods". Incorporation of isotopes through the addition of labeled amino acids significantly reduces the background signal from lipids, but it does not reduce the incorporation of isotopes into endogenous proteins. Although the background signal will be less than in a uniformly labeled sample, as fewer amino acids are labeled, use of rifampicin will still block the incorporation of labeled amino acids into endogenous proteins. Two YidC cellular membrane samples were prepared with different amino acid combinations, chosen to avoid unintentional labeling of additional amino acids via metabolic scrambling. In these specifically labeled samples, background lipid signals were negligible, and ^13^C–^13^C correlation experiments could be used without ^15^N-editing steps (Fig. 4). In the threonine and isoleucine labeled sample (Fig. 4a and cutouts in Fig. 4b, c), 73 out of 548 residues were labeled (~13 %), while in the methionine and arginine labeled sample (Fig. 4d with cutouts in Fig. 4e, f), 34 were labeled (~6 %). For both samples, sensitivity was sufficient for a two-dimensional ^13^C–^13^C correlation CP-based experiment with 30 or 150 ms PARIS mixing to be recorded with less than 300 scans per t_1_ point. The spectra exclusively contained correlations expected for intra-residue atoms separated by 1 or 2 bonds (Thr, Ile, Fig. 4a, b) and (Met, Arg, Fig. 4d with cutout e, f). The observed resonance frequencies agreed with a spectrum obtained on uniformly labeled YidC proteoliposomes (Fig. 4, grey), and they were in line with predicted correlations using FANDAS (Gradmann et al. 2012) derived from the recent crystal structure of YidC (PDB ID 3wvf, (Kumazaki et al. 2014). The decrease in spectral crowding was significant.

**Fig. 5:**
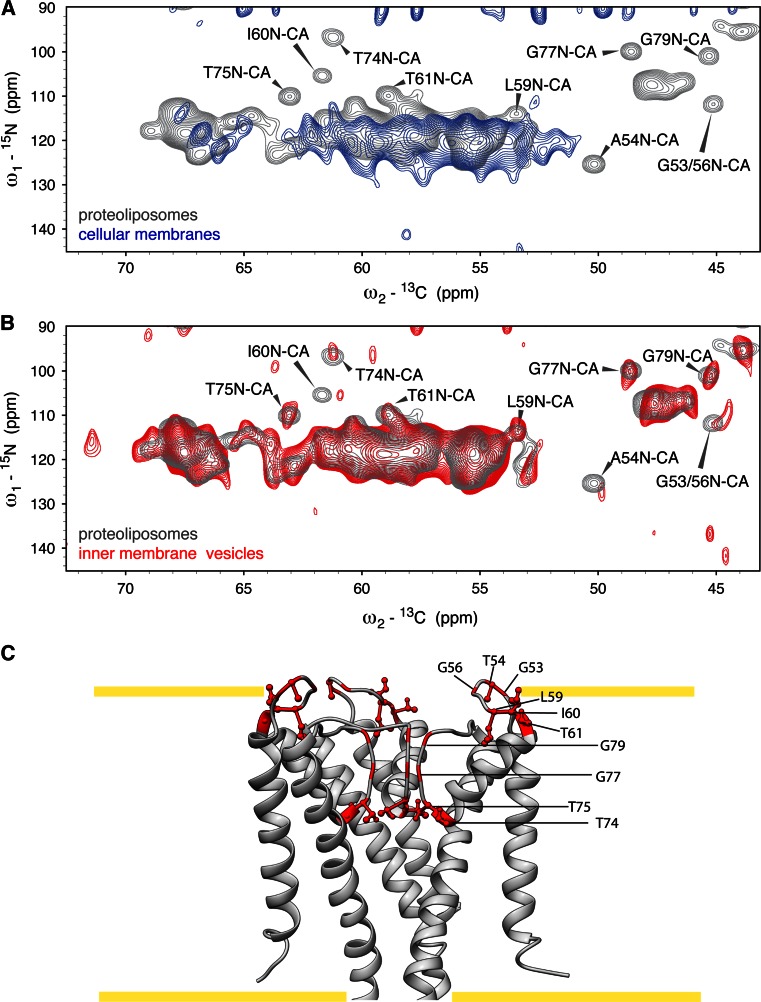
Separation of inner and outer membranes improves the sensitivity of cellular membrane samples. ^15^N–^13^C_α_ CP-based correlation spectra of KcsA in cellular membranes (**a** *blue*) and inner membrane vesicles (**b** *red*) prepared with rifampicin, in comparison to purified and reconstituted KcsA (**a**, **b** *grey*). Each spectrum was recorded with a 350 μs increment to 6 ms total acquisition time in the indirect dimension and 8 ms total acquisition in the direct, averaged over 1072 scans, and processed after zero-filling without linear prediction with QSINE 3 and 2.5 in the direct and indirect dimensions, respectively. In **b**, previously assigned resonances (Schneider et al. 2008; van der Cruijsen et al. 2013) that are functionally important and resolved in the inner membrane samples are indicated. The positions of the indicated residues with assigned resonances are indicated in *red* on the structure of KcsA (PDB 3EFF) in **c**, where the approximate membrane boundaries are shown as *yellow lines*. One subunit of KcsA is not shown for clarity

To investigate whether rifampicin-based repression of endogenous expression allows for successful preparation of cellular membrane samples with MPs that have significantly lower expression levels than YidC, we prepared fully isotopically labeled cellular membrane samples for the potassium channel KcsA from *S. lividans*. As shown in Fig. 1b, in our expression system KcsA expresses in significantly lower amounts than both YidC and the endogenous outer membrane proteins (designated as OMP in Fig. 1), which are typically present in *E. coli* cells at copy numbers of ~10^5^/cell (Renault et al. 2012b, references therein). In Fig. 5a, we compare results of ^15^N–^13^C_α_ correlation experiments conducted on uniformly labeled cellular envelopes containing over-expressed KcsA (blue) to data obtained using reconstituted (^13^C,^15^N) labeled KcsA in asolectin liposomes for which assignments are available (Schneider et al. 2008; van der Cruijsen et al. 2013). Figure 5a suggests that cellular envelope preparations are dominated by other protein components, potentially stemming from outer membrane proteins such as OmpA/F, and LPP (outer membrane *l*i*p*o*p*rotein) that endogenously express to high levels (Stenberg et al. 2005; Renault et al. 2012b, and references therein). The resonances observed in the KcsA cellular membrane samples (Fig. 5a) are different to those observed in the YidC cellular membrane samples (Fig. 2a), as demonstrated in Supplementary Fig. 1a. Indeed, the observed ^15^N–^13^C_α_ correlation pattern from cellular envelope membranes (Fig. 5a, blue) shows striking overlap with that of purified Lpp (see Supplemental Figure 1B).

To remove signal from outer membrane proteins, we explored the use of inner membrane vesicles prepared from our cellular envelope samples. The sucrose gradient used to separate the inner and outer membrane resulted in a relatively low yield of inner membranes when cells were grown in M9 media, so ~200 mL of cell culture at OD ~2.0 was needed to fill a 3.2 mm MAS rotor. However, the spectral quality of ^15^N–^13^C_α_ SPECIFIC CP-based experiments using inner membrane samples of KcsA was considerably improved compared to using cellular membrane samples (Fig. 5b). We could readily observe resolved peaks with previous assignments (Schneider et al. 2008; van der Cruijsen et al. 2013) to the turret region (such as T61) and selectivity filter (T74, T75 or G79) of KcsA in the fully labeled inner membrane samples (Fig. 5b, c). Interestingly, we observed reduced ssNMR intensities for T74, for which we have recently found increased dynamics in the closed-conductive state (Koers et al. 2014). The same applies to G53 and A54, which exhibit stronger dynamics compared to other regions of the turret, such as T61 (van der Cruijsen et al. in preparation). Given that the lipid character influences the function of KcsA channels in lipid bilayers (Weingarth et al. 2013), these data provide initial evidence for structural conservation of KcsA irrespective of whether membrane mimetics such as asolectin that is rich in the zwitterionic phosphatidyl choline (PC) or *E. coli* inner membranes (rich in zwitterionic phosphatidyl ethanolamine, PE) are used.

## Conclusions

Studying complex molecules in their biological context is an exciting prospect for structural biology. There is increasing evidence that the environment is an important contributor to the biophysical properties of proteins, modulating important biological processes such as ligand binding (e.g. Hubbard et al. 2003) and protein–protein interactions (e.g. Burz et al. 2006). In-cell NMR has even revealed different protein folding states in vitro and in vivo (Dedmon et al. 2002). For MPs, the impact of different environmental mimics has been well established in vitro. The difference between in vitro and in vivo experiments, however, is less well understood, due to the challenges of probing biological processes at an atomic level in a specific manner for these proteins. The work presented here expands the scope of cellular ssNMR to study the properties of MPs in their native environment. We have demonstrated that rifampicin treatment can be used to produce cellular membrane samples for ssNMR with specific protein labeling. Unlike in-cell NMR experiments where selective isotope incorporation was not found to significantly improve ^15^N experiments (Serber et al. 2001), we have demonstrated a clear improvement in ssNMR spectra of cellular membrane samples upon removal of background signals with rifampicin. This difference is most likely due to the use of direct ^13^C detection in our ssNMR experiments. These samples can be adapted to different isotopic labeling schemes and protein expression levels. As demonstrated with KcsA, individual resonances, important for function, can be observed. Although the rifampicin treatment can only be used to inhibit bacterial RNA polymerases (Campbell et al. 2001), a method similar to the single protein production (SPP) system (Suzuki et al. 2005) could potentially be used with eukaryotic cells.

Expression, purification and reconstitution of MPs into synthetic lipid bilayers can be a slow process, often requiring several weeks for more complicated samples. Purification necessitates removing MPs from their native environment, which potentially induces structural changes and loss of activity. Some membrane proteins are not amenable to established purification methods, and nearly impossible to purify in large quantities. As such, any method that allows MPs to be characterized in their native environment not only augments the biological relevance of any observations, but also presents an opportunity to study previously intractable systems. This system also naturally lends itself to the study of MP complexes, which are often disrupted by detergent solubilization.

From a spectroscopic point of view, rifampicin-treated cellular membrane samples present some unique advantages. Assignment of resonances in MPs can be challenging and dedicated labeling schemes are usually needed to reduce spectral overlap (see, for example, Etzkorn et al. 2007; Shi et al. 2011; Wang et al. 2013b; Sinnige et al. 2015). Specific labeling of a small number of amino acids presents an additional means to aid ssNMR assignment for these larger MPs. Due to the large number of samples that must be prepared to exhaustively cover the protein, this method would be extremely time and resource intensive for purified proteins. Here we have demonstrated that it is possible to study MPs isotopically labeled at a small number of amino acids from small cell culture volumes (~50 mL) without purification. This system does not rely on concentrating large cell cultures into small volumes before induction (as done in Mao et al. 2011), which in our hands results in lower expression levels of both membrane and soluble proteins. The rifampicin system presents a method by which to employ specific amino acid labeling quickly and inexpensively as an assignment strategy, potentially allowing ssNMR studies of membrane proteins which cannot be purified, of large membrane proteins with significant spectral overlap, and of membrane protein complexes.

## Electronic supplementary material

Supplementary material 1 (DOCX 331 kb)
