# Supplementary material for: Efficient cellular solid-state NMR of membrane proteins by targeted protein labeling
Source: J Biomol NMR. 2015 May 9;62(2):199–208. doi: 10.1007/s10858-015-9936-5 (PMC4451474; doi:10.1007/s10858-015-9936-5)
Supplement: Supplementary file 1 — Supplementary material 1 (DOCX 331 kb) [file 10858_2015_9936_MOESM1_ESM.docx]

**Supporting information:**


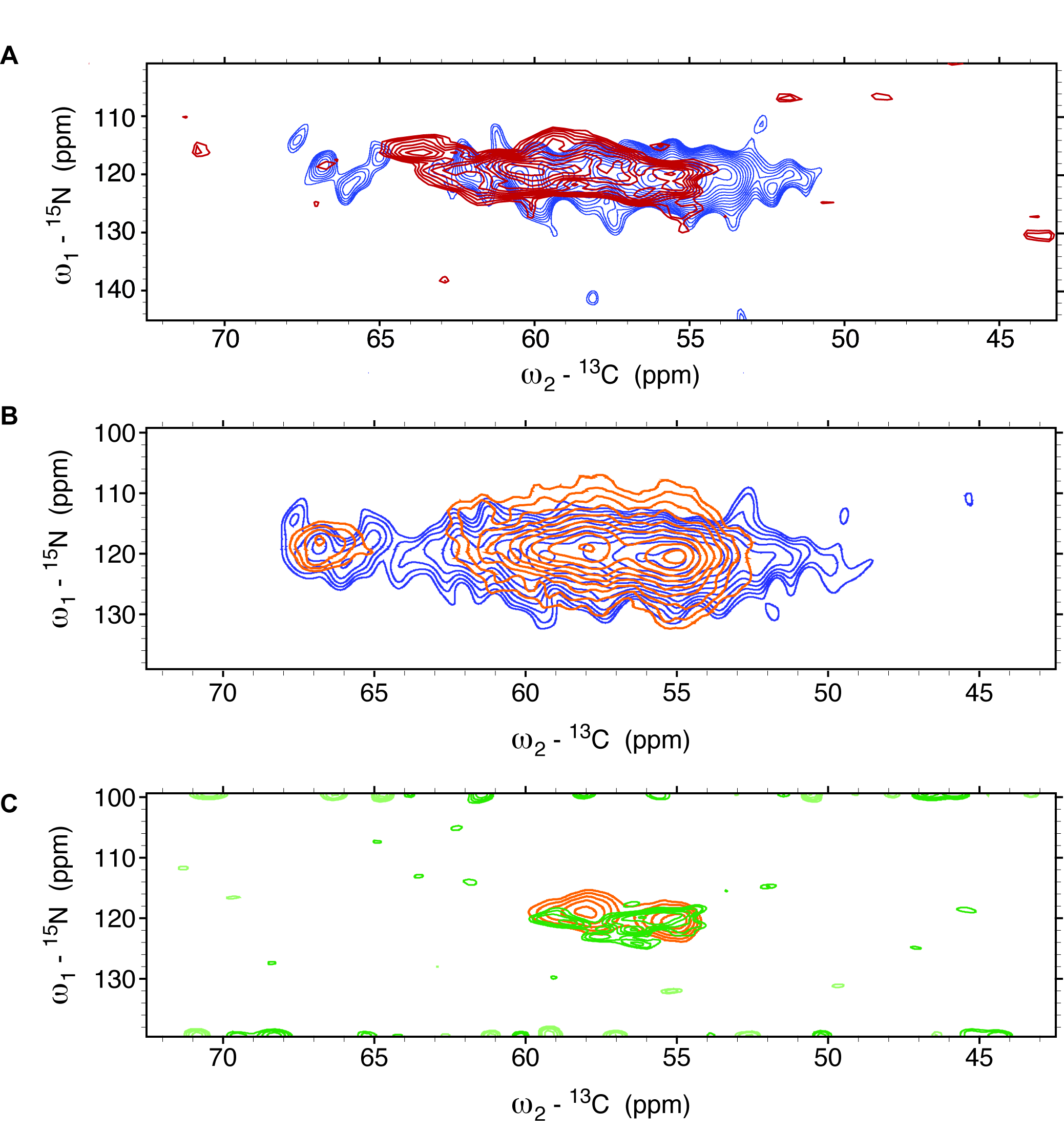


Supplementary Figure 1: Signals from the naturally abundant outer membrane lipoprotein LPP, which exists at copy numbers ~ 10^5^ for *E. coli*, could be responsible for features observed in spectra of KcsA cellular membranes. A) There is a significant difference between the cellular membrane sample of YidC (red) and KcsA (blue) in^15^N-^13^C_α_  CP-based correlation spectra. B) Overlay of ^15^N-^13^C_α_  CP-based correlation spectra of purified LPP in orange (Kaplan *et al.*, unpublished results) and KcsA cellular membranes in blue (as shown in Figure 5A). C) The same spectrum of LPP as in A), except drawn at higher contour levels in orange, overlaid with the spectrum from Figure 2C, of YidC cellular membranes produced with rifampicin, but without protein over-expression (-IPTG) in dark green. Light green represents negative contours in the –IPTG data.
